# Building the Positive Emotion-Resilience-Coping Efficacy Model for COVID-19 Pandemic

**DOI:** 10.3389/fpsyg.2022.764811

**Published:** 2022-03-17

**Authors:** Guek Nee Ke, Dasha Grajfoner, Rachel Mei Ming Wong, Stephen Carter, Rozainee Khairudin, Wee Yeap Lau, Khalil Anwar Kamal, Shen Chieng Lee

**Affiliations:** ^1^Department of Psychology, School of Social Sciences, Heriot-Watt University Malaysia, Putrajaya, Malaysia; ^2^Department of Psychology, School of Social Sciences, Heriot-Watt University Edinburgh, Edinburgh, United Kingdom; ^3^Edinburgh Business School, School of Social Sciences, Heriot-Watt University Edinburgh, Edinburgh, United Kingdom; ^4^National University of Malaysia, Bangi, Malaysia; ^5^Faculty of Economics and Administration, Department of Applied Statistics, University Malaya, Kuala Lumpur, Malaysia; ^6^Malaysian Institute of Economic Research (MIER), Kuala Lumpur, Malaysia

**Keywords:** COVID-19, psychological wellbeing, positive emotion, resilience, coping self-efficacy, depression

## Abstract

The world faces unprecedented challenges because of the Coronavirus Disease 2019 (COVID-19). Existing theories of human flourishing and coping efficacy are too broad and general to address COVID-19 unprecedented mental health challenges. This study examined two main objectives, first the associations between psychological outcomes (i.e., depression, anxiety, and stress) and psychological wellbeing of this phenomenon, and second, moderating and mediating factors emotions, resilience and coping self-efficacy. A nationwide survey was carried out on a Malaysian sample (*n* = 920). Participants completed an on-line survey that assessed psychological outcomes, psychological wellbeing, positive–negative emotions, resilience, and coping self-efficacy. The relationship between psychological states and psychological wellbeing was successfully mediated by coping self-efficacy (direct effects of −0.31 to −0.46 at *p* < 0.01) and resilience (direct effects of −0.06 to −0.26 at *p* < 0.01). Moreover, positive emotion significantly moderated depression (*b* = −0.02, *p* < 0.01) and anxiety (*b* = −0.14, *p* = 0.05) with psychological wellbeing. Findings highlighted the importance of these factors in developing a dedicated model to be built into the recovery plan to ameliorate the negative impact of COVID-19 on psychological wellbeing. Hence, the Positive Emotion-Resilience-Coping Efficacy Model was developed.

## Introduction

Discovery of the Coronavirus 2019 (COVID-19) in 2019, has created an adverse impact on the physical and mental health of people around the world. Research across the globe has been focusing on, and aiming to understand, the current and future adverse impact. As COVID-19 is one key issue, another equally important issue is public intervention such as a lockdown. Countries in the world, including Malaysia, have implemented lockdowns to curb the spread of the virus at the community level (Minhat and Kadir Shahar, 2020). The Malaysian government implementation of the Movement Control Order (MCO), or Lockdown, has been recognized as an effective public health intervention in curbing the country’s COVID-19 outbreak (Shah et al., 2020; Tang, 2020). Although essential, such drastic interventions have contributed to adverse psychological outcomes such as depression, anxiety, and stress (Cao et al., 2020; Zhang et al., 2020). Existing COVID-19 research in Malaysia has been predominantly non-empirical and report the repercussions of the MCO on the corporate sector, medical industry, and the environment (Lip et al., 2020; Naderipour et al., 2020; Waiho et al., 2020). The direction of current research presents an important research gap which this paper intends to fill: the lack of empirical data on the psychological impact of COVID-19 on a significant sample of Malaysians, and potential recovery strategies that incorporate positive psychological interventions. Past research, in various contexts, has hypothesized that the positive psychological constructs such as positive emotion, resilience, and coping self-efficacy would significantly moderate and mediate the relationship between depression, anxiety, and stress with psychological wellbeing. Furthermore, the potential recovery intervention techniques that integrate positive and coaching psychology are also discussed, given their efficacy in improving psychological wellbeing (Palmer et al., 2020; Song et al., 2020). Drawing on non-clinical psychological theories, the Theory of Positive Psychology and the Theory of Coping and Self-Efficacy, this study aims to not only investigate the psychological impacts but also how resilience, self-efficacy, and positive emotions might mitigate these impacts brought by COVID-19. For the afore-mentioned reasons, a wider diversity of the population was drawn in this study.

Numerous methods have been used in various study groups to mitigate COVID-19 related psychological and mental health issues. These methods include animal accompanied (Grajfoner et al., 2021), spiritual and telehealth for palliative care (Palmer et al., 2020), network building intervention (Nooraie et al., 2021), Yoga (Sahni et al., 2021) and others. However, a purposeful model for COVID-19 based on positive psychology core values has not been established.

## Literature Review

Firstly, the following literature review is intended to further understand the relationship between psychological domains and psychological wellbeing, with potential intervention, applying positive psychology core values.

Studies, in a global context, report an elevation in the domains of depression, anxiety, and stress as direct psychological consequences of COVID-19 (Asmundson and Taylor, 2020; Li et al., 2020; Salari et al., 2020), reflecting previous findings on other pandemics (e.g., 2003 SARS, 2009 H1N1; see Hawryluck et al., 2004; Wheaton et al., 2012). Moreover, these psychological conditions are exacerbated by the increase in negative emotions because of the severity of not only the outbreak, but the lockdown implemented to contain it (Brooks et al., 2020; Lades et al., 2020; Wang et al., 2020). Experiencing negative emotions not only impedes the maintenance of a healthy psychological wellbeing, but evidence suggests that this prevalence has long-term implications on an individual and community level, including being predictors of serious psychological and physiological conditions (Emdin et al., 2016; Czeisler et al., 2020; Restubog et al., 2020). Research presents the possibility that negative emotions are associated with self-protective behaviors, and that negative and positive emotions are mutually exclusive (Cheung and Tse, 2008; Wang et al., 2020). These opposing perspectives present a unique conundrum, where the alleviation of negative emotions may not be dependent on positive emotions, nor are necessary for the improvement of self-protective behaviors. Nonetheless, it can be established that a reduction in negative emotions through the perception of control or institutional trust during a time of crisis (Cheung and Tse, 2008; Yang and Ma, 2020) may be beneficial for psychological wellbeing. Hence, it is posited that the prevalence of both negative and positive emotions may serve as a moderator when investigating the relationship between psychological outcomes and psychological wellbeing.

Relatedly, positive psychology explains that these psychological domains (i.e., depression, anxiety, and stress) are threats to one’s sense of wellbeing, an important element in the capacity to flourish in everyday life (Seligman, 2011). The current debate illustrates that rather than indulging in a pessimistic outlook on life post-COVID-19, an individual is recommended to focus on eudaimonia, a core value in positive psychology that idealizes a well-lived and fulfilled life (Seligman and Csikszentmihalyi, 2014). Positive psychological interventions prove beneficial in the long-term, as negative events are unavoidable in life and one’s adaptive (or maladaptive) response determines the behavior toward the circumstances. Past research on mitigation and intervention often refer to approaches underpinning psychology models and elements. Among the most referred and applied theories under adverse situation are the Theory of Positive Psychology (PERMA Model-Seligman, 2011), the Theory of Coping (Folkman and Lazarus, 1984) and the Theory of Self-Efficacy (Bandura, 1982). This study focused on three positive psychology elements derived from these models: resilience, self-efficacy, and positive emotions. By promoting positive psychological elements, the individual learns to be content with one’s past, remaining happy in the present, and being hopeful for the future. The relevancy of such suggestion is proven, given that COVID-19 has been far from completely eradicated, and the transition to the vastly different ‘new normal’ is seen as an imperative measure to contain the spread of the virus (Jamaludin et al., 2020).

By focusing on positive psychology in more detail, it is relevant to refer to, and elaborate on, Seligman’s (2018) PERMA model which consist of five core domains namely positive emotion; engagement; relationships; meaning, and accomplishment. This model provides a framework that is not soley contingent on happiness but on the proactive development of each component to not only improve aspects of wellbeing, but decrease one’s psychological distress (Forgeard et al., 2011; Ascenso et al., 2018; Farmer and Cotter, 2021). Contextually, positive emotion encompasses a few core values and top of the list is happiness, followed by gratitude, love, optimism, and contentment (Seligman, 2011). Research on positive emotion links its increment with the ability to improve reactions to negative stimuli, and promote resilience (Tugade et al., 2004). Positivity in the face of a crisis develops resilience, i.e., one’s ability to cope with adversity (Grant and Kinman, 2014). Having established positive emotion, it is possible to consider resilience as another mitigation (see Bolton et al., 2017). The veracity of resilience has been noted in a variety of contexts and social groups (Longstaff and Yang, 2008; Barzilay et al., 2020). Enhancing resilience has been associated with better mental wellbeing, along with bidirectional improvements in psychological outcomes such as self-efficacy, positive emotion, and coping (Lee et al., 2013; Robertson et al., 2015; Liu et al., 2018). In the case of COVID-19, individual and shared resilience is vital when overcoming a global pandemic, especially in one as severe as COVID-19 (Lazzarino et al., 2014; Prime et al., 2020; Vinkers et al., 2020). Elcheroth and Drury (2020) further identify collective resilience as the key to socially effective responses to the pandemic, which substantiates this study’s emphasis on resilience as another mitigating factor to ameliorate the impact of the pandemic on psychological wellbeing.

The complementary role of coping self-efficacy in a few psychological theories, such as the Theory of Coping by Folkman and Lazarus’s (1984) and Bandura’s (1982) Theory of Self-Efficacy, is another important element investigated. Self-efficacy is extended from the traditional definition by Bandura (1982), in that coping self-efficacy specifically addresses one’s confidence in the ability to cope effectively when facing challenges. Here, it serves as a pivotal prerequisite to either maladaptive (i.e., coping that fails to regulate distress or manage the underlying problem) or adaptive coping behavior (e.g., Bidzan et al., 2020). Hence, when taken in conjunction with resilience in this study, coping self-efficacy can be described as a source of improvement for resilience, as one’s belief in one’s own ability to cope (i.e., coping self-efficacy), allows one to return to pre-crisis status quickly through the implementation of coping strategies. The multiple avenues of application of coping self-efficacy also makes it an effective component in prevention and intervention models that would improve psychological wellbeing, especially after a crisis. Emphasis on the development of these skills on an individual level is influenced by the social distancing practices implemented to manage COVID-19.

### Hypotheses

**Figure 1:**
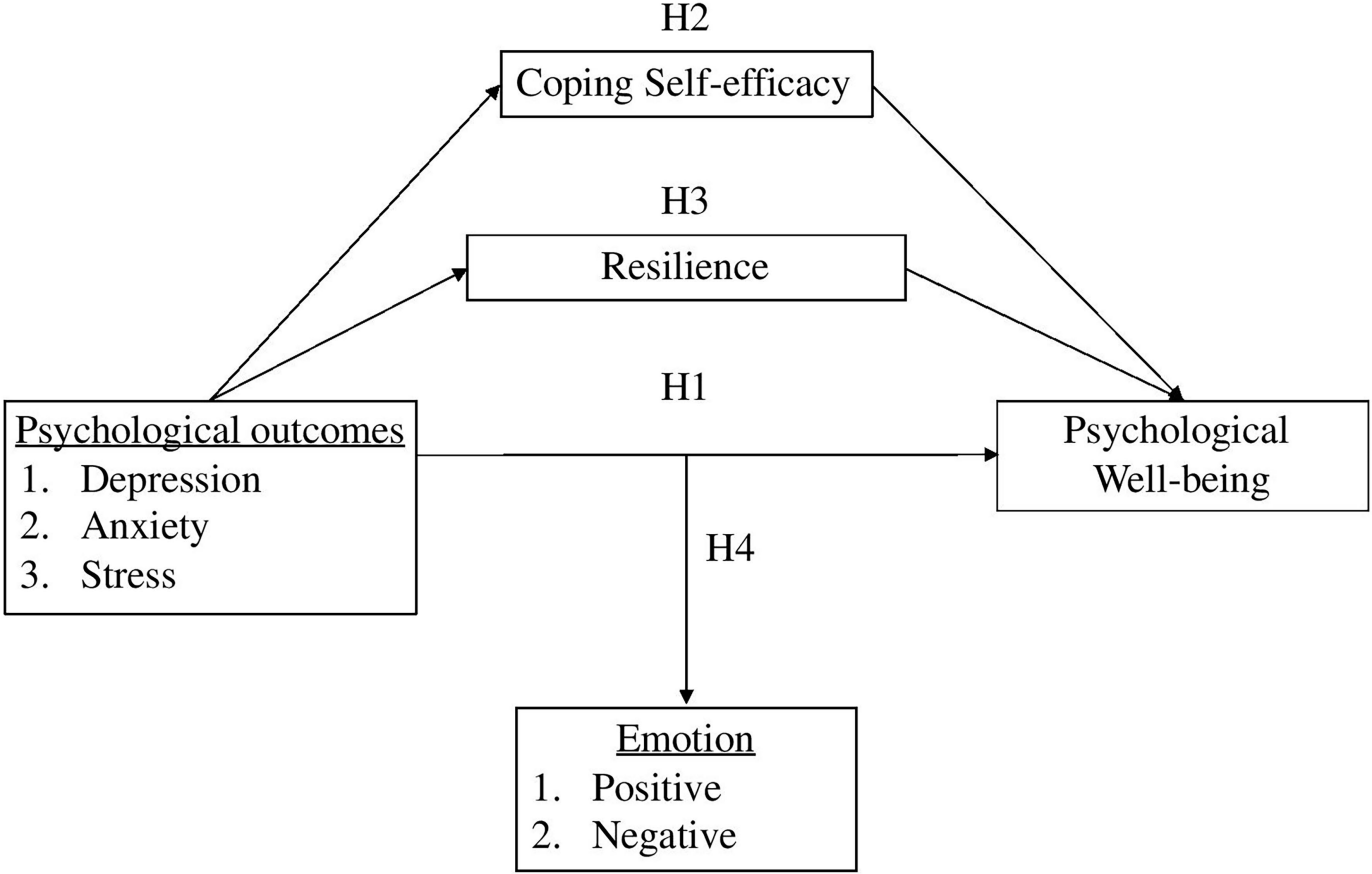
The conceptional framework of the mediation-moderation model. Note: independent variable (IV): depression, anxiety, stress; dependent variable (DV): psychological wellbeing; mediator: resilience, coping self-efficacy; moderator: Positive emotion.

So far, evidence highlights that the core values of positive psychology and coping-efficacy mitigate and promote better psychological wellbeing. Hence, this study focuses on three elements; positive emotion, resilience and coping self-efficacy, which are the key components in a flourishing everyday life. The current study extends the scope of these findings into related but distinct contexts of the novel COVID-19 pandemic. Thus, the study proposed a conceptual framework (Figure 1) and the following hypotheses are tested:

*H1*: Depression, anxiety, and stress associate negatively with psychological wellbeing.

*H2*: Emotion moderates the relationship between depression, anxiety, and stress with psychological wellbeing.

*H3*: Resilience mediates the relationship between depression, anxiety, and stress with psychological wellbeing.

*H4*: Coping self-efficacy mediates the relationship between depression, anxiety, and stress with psychological wellbeing.

### Significance of the Study

Given the severity of COVID-19 and its’ long-lasting impact on society, it is important to understand the psychological impact of this health crisis on a community and individual level to address it effectively. The findings of this study will add to existing literature of COVID-19 from the perspective of a Southeast Asian country and reveal the extent to which the psychological variables react with each other. Moreover, the results will provide practical insights into formulating effective public health interventions that are both reactive for the current COVID-19 crisis and proactive for any future health crisis.

## Methods

### Participants

The sample size was estimated using a single proportion formula (Daniel, 1999) based on Malaysia’s population size 32.7 million (estimated by the Department of Statistics Malaysia) at the time of the study and a 95% confidence level. A total of 1,217 responses were collected, but those that did not provide sufficient data on the measures of interest were considered invalid and excluded from analysis. The method employed to identify invalid responses was SPSS’ Descriptive and Frequency functions, where participants who did not complete the survey were removed entirely. After considering this criterion, 297 responses were excluded from the analysis, leaving a final sample size of 920, which exceeded the recommended size. Participants completed the survey during the implementation of Malaysia’s Movement Control Order (MCO). Participants were between the ages of 18–82 (*M* = 35.25, SD = 11.86), 54.5% were male (*n* = 501) and 45.5% were female (*n* = 419). Table 1 summarizes the participant’s characteristics.

**Table 1:** Demographic characteristics of the participants (*n* = 920).

Variables	*n*	%
Gender		
Male	419	45.5
Female	501	54.5
Age		
18–35	530	57.6
36–53	309	33.6
>54	81	8.8
Educational level		
No formal education	10	1.1
High school or secondary school	67	7.3
Undergraduate or college	705	76.6
Master’s degree or above	138	15.0
Monthly income		
<RM 1,000.00	119	12.9
RM 1,001.00–RM 2,000.00	93	10.1
RM 2,001.00–RM 3,000.00	120	13.0
RM 3,001.00–RM 4,000.00	130	14.1
RM 4,001.00–RM 5,000.00	88	9.6
RM 5,001.00–RM 7,500.00	132	14.3
RM 7,501.00–RM 10,000.00	97	10.5
>RM 10,001.00	141	15.3

### Procedure

The cross-sectional sampling frame included adults from all 13 states and three federal territories in Malaysia. The survey was available in two languages (English and Bahasa Malaysia) to encourage participation from non-English speakers. Data collection was conducted nationwide between June to July 2020. In consideration of COVID-19 health and safety measures, participation was done digitally (Qualtrics) to adhere to physical distancing requirements. Access to the survey was through a QR code on posters strategically located in shopping malls in various states. Professional associations, NGOs, industry partners and government agencies were also contacted through email for questionnaire distribution.

Participants were initially introduced to the broad aims of the study in the information sheet at the beginning of the survey. A consent form reminded participants of their rights, which included assurance of anonymity and the right to withdraw at any point. Once consent was provided, they were automatically directed to the start of the survey. In addition to demographic questions, a total of five psychological measures were used to measure wellbeing, emotions, psychological states, resilience, and coping self-efficacy. A debrief sheet was made available at the end of the survey to provide further information about the study and additional resources for the participants.

### Materials

*The Warwick-Edinburgh Mental wellbeing Scale* (WEMWBS; Tennant et al., 2007) was used to measure mental wellbeing. It comprises 14 positively phrased Likert-style items and fulfills the classic criteria for scale development. For our sample, the WEMWBS (*M* = 44.15, SD = 10.55) had excellent reliability at *α* = 0.93. This is consistent with the scale’s original reliability (*α* = 0.91) and Faudzi et al.’s (2019) reported reliability of *α* = 0.90.

*The Depression, Anxiety, and Stress* (DASS-21; Henry and Crawford, 2005) is a psychometric scale that assesses symptoms of depression, anxiety, and stress. This study used the shorter, 21-item version that has been validated in multiple studies (Oei et al., 2013). The reliability of the DASS-21 in this sample ranged from good to excellent, *α*_Overall_ = 0.96, *α*_D_ = 0.91, *α*_A_ = 0.88, *α*_S_ = 0.89. The overall reliability was consistent with the original *α* = 0.93 and *α* = 0.95 reported by Ng et al. (2014).

*The Positive and Negative Affect Schedule* or (PANAS; Watson et al., 1988) is a 20-item scale that measures the participants affective experiences. In this study, the PANAS (*M*_PA_ = 30.30, SD_PA_ = 7.60; *M*_NA_ = 25.29, SD_NA_ = 7.70), had good to excellent reliability at *α*_overall_ = 0.86, *α*_PA_ = 0.91, and *α*_NA_ = 0.90. This is consistent with the scale’s original reliability (*α*_PA_ = 0.88, and *α*_NA_ = 0.87) and exceeds (Omar et al., 2020) reported reliability of *α*_PA_ = 0.77, and *α*_NA_ = 0.84.

*The Brief Resilience Scale* (BRS; Smith et al., 2008) uses 6-items to measure an individual’s ability to bounce back, resist illness, adapt to stress or thrive in the face of adversity. The BRS (*M* = 3.16, SD = 0.55) had adequate reliability at *α* = 0.57 for the chosen sample, compared to the Cronbach alpha ranging from 0.80–0.91 of the original scale. Previous studies have suggested that this value is acceptable for scales with a small number of items (Streiner, 2003; Morera and Stokes, 2016).

*The Coping-Self Efficacy* (CSE; Chesney et al., 2006) provides a measure of a person’s perceived ability to cope effectively with life challenges, as well as a way to assess changes in CSE over time in intervention research. It comprises 26 Likert-style items, and the CSE (*M* = 172.94, SD = 35.64) had a reliability of *α* = 0.95 in this study, consistent with the original study’s reliability of *α* = 0.95. All questionnaires used were established questionnaires with a good level of reliability.

### Statistical Analysis

The data collected was analyzed using PROCESS MACRO in SPSS. Given that there were no latent variables being measured, any differences were considered inconsequential (Hayes et al., 2017).

## Results

### Demographic Characteristics

Cross-tabulation analysis was executed using SPSS to identify any possible associations between participant characteristics (i.e., income groups, age, gender, education) and the dependent variables (i.e., depression, anxiety, stress, psychological wellbeing, resilience, coping self-efficacy, positive and negative emotions). A summary of the mean differences is presented in Table 2. The significant main findings indicate that income has the most significant influence on almost all variables, followed by age, gender, and lastly, education.

**Table 2:** Summary of mean differences between demographic and dependent variables.

Variables	Gender (*n*)		Age (*n*)			Education (*n*)				Income (*n*)							
	Male (419)	Female (501)	18–35 (530)	36–53 (309)	>54 (81)	1 (10)	2 (67)	3 (705)	4 (138)	1 (119)	2 (93)	3 (120)	4 (130)	5 (88)	6 (132)	7 (97)	8 (141)
Psychological outcome																	
Depression	23.75	24.49	25.87^**^	22.17^**^	20.44^**^	26.60	25.16	24.27	22.90	25.50^*^	26.13^*^	25.40^*^	23.80^*^	24.73^*^	22.53^*^	22.49^*^	23.28^*^
Anxiety	22.27	22.78	23.94^**^	21.18^**^	18.69^**^	27.40	23.82	22.56	21.52	24.17^**^	24.37^**^	23.98^**^	22.46^**^	23.59^**^	21.14^**^	21.11^**^	20.50^**^
Stress	24.49	25.14	26.13^**^	23.56^**^	21.31^**^	26.40	25.58	24.95	23.80	25.71	25.74	26.32	24.62	26.05	23.36	23.59	23.96
Psychological wellbeing	44.97^*^	43.46^*^	43.31^*^	45.01^*^	46.37^*^	38.90^**^	38.79^**^	44.37^**^	46.02^**^	42.25^**^	40.26^**^	41.18^**^	43.34^**^	42.88^**^	46.61^**^	47.84^**^	47.55^**^
Resilience	19.41^**^	18.60^**^	18.50^**^	19.47^**^	20.14^**^	18.40^**^	18.52^**^	18.91^**^	19.50^**^	18.14^**^	18.32^**^	18.19^**^	18.50^**^	18.53^**^	19.43^**^	19.56^**^	20.61^**^
Coping self-efficacy	86.10	86.78	85.24^*^	88.13^*^	88.20^*^	66.61	79.99	86.85	89.11	85.41^**^	78.35^**^	84.31^**^	84.87^**^	84.65^**^	88.60^**^	89.59^**^	93.02^**^
Emotion																	
Positive	30.95^*^	29.76^*^	30.02	30.61	30.93	24.00^**^	27.70^**^	30.33^**^	31.86^**^	29.79^**^	28.30^**^	28.32^**^	29.78^**^	29.65^**^	31.70^**^	31.91^**^	32.21^**^
Negative	25.03	25.51	26.25^**^	24.40^**^	22.44^**^	23.80	25.97	25.41	24.45	25.99^**^	25.58^**^	27.36^**^	26.18^**^	25.10^**^	24.80^**^	24.39^**^	23.13^**^

* *p* < 0.05;

** *p* < 0.01.

Educational level: (1). No formal education (2). High school or secondary school (3). Undergraduate or college (4). Master’s degree or above; monthly income: (1). <RM 1,000.00; (2). RM 1,001.00–RM 2,000.00; (3). RM 2,001.00–RM 3,000.00; (4). RM 3,001.00–RM 4,000.00; (5). RM 4,001.00–RM 5,000.00; (6). RM 5,001.00–RM 7,500.00; (7). RM 7,501.00–RM 10,000.00; (8). >RM 10,001.00. Score categories: Psychological outcomes: depression, stress, anxiety (range: 14–56; Low = <23, Moderate = 24–37, High = > 38). Psychological wellbeing (range: 14–70; Low = <34, Moderate = 35–54, High = > 55). Resilience (Range: 6–30; Low = <14, Moderate = 15–23, High = > 24). Coping self-efficacy (Range: 26–130; Low = <60, Moderate = 61–95, High = > 96). Emotions (range: 10–50; Low = <23, Moderate = 24–37, High = > 38).

#### Income

One-way ANOVA indicated that significant differences were observed between income groups, particularly in depression [*f* (7, 912) = 2.40, *p* = 0.020], anxiety [*f* (7, 912) = 3.88, *p* < 0.001], psychological wellbeing [*f* (7, 912) = 9.37, *p* < 0.001], resilience [*f* (7, 912) = 9.49, *p* < 0.001], coping self-efficacy [*f* (7, 912) = 7.08, *p* < 0.001], positive [*f* (7, 912) = 5.02, *p* < 0.001] and negative emotions [*f* (7, 912) = 3.57, *p* < 0.001]. Predictably, these results suggest that participants with higher incomes were able to cope better with the lockdown, due to their improved access to resources, compared to those with lower incomes. This pattern is also reflected in Pieh et al.’s (2020) study in an Austrian sample and can be ascribed to the economic impact of COVID-19 and the resulting lockdown on earning ability.

#### Age

Subsequent analysis between age groups identified significant differences when comparing depression [*f* (2, 917) = 22.06, *p* < 0.001], anxiety [*f* (2, 917) = 19.20, *p* < 0.001], stress [*f* (2, 917) = 14.71, *p* < 0.001], psychological wellbeing [*f* (2, 917) = 4.53, *p* = 0.011], resilience [*f* (2, 917) = 14.47, *p* < 0.001], coping self-efficacy [*f* (2, 917) = 3.01, *p* = 0.050], and negative emotions [*f* (2, 917) = 11.99, *p* < 0.001]. Consistent with Benke et al.’s (2020) findings, younger adults were more mentally vulnerable than older adults during lockdown. This is likely due to the disruption of social life due to lockdown orders (Asselmann and Specht, 2020; Luchetti et al., 2020), and improved resilience in older adults (Chen et al., 2018).

#### Gender

An independent sample *t*-test between gender revealed a significant difference with regards to psychological wellbeing [*t* (918) = 2.17, *p* = 0.030], resilience [*t* (918) = 3.73, *p* < 0.001], and positive emotion, [*t* (918) = 2.37, *p* = 0.018]. The gender difference between males scoring higher than females on the stated variables is reflective of both labor and cognitive gaps as suggested by Farré et al. (2020), whereby women are more likely to be burdened with additional household responsibilities (e.g., childcare), which leads to a reduction in work hours and hence income (Czymara et al., 2021; Dang and Nguyen, 2021).

#### Education

Relatively less important, was the participant characteristic of “education,” with significant differences only observed in psychological wellbeing [*f* (3, 916) = 8.32, *p* < 0.001], coping self-efficacy [*f* (3, 916) = 8.41, *p* < 0.001], and positive emotion [*f* (3, 916) = 6.99, *p* < 0.001]. It is likely that the more educated an individual is, the more receptive they are to the knowledge of protective factors such as coping self-efficacy and positive emotion that promote wellbeing. Nonetheless, as the sample size for each education level was not proportionate, it may have impacted the significance of these differences.

### Correlation and Regression Analysis

Preliminary statistical analysis of the correlation between depression, anxiety, and stress with psychological wellbeing, are summarized in Table 3. Correlations were significant and in the expected direction, indicating that any increase in depression, anxiety, and stress would result in a decline in significant psychological wellbeing.

**Table 3:** Descriptive statistics and correlations among the key variables.

	Variables	*M*	SD	1	2	3	4
1	Psychological wellbeing	44.15	10.55	–			
2	Depression	24.15	9.60	−0.29^**^	–		
3	Anxiety	22.55	8.72	−0.23^**^	0.85^**^	–	
4	Stress	24.84	9.16	−0.17^**^	0.86^**^	0.85^**^	–

** Correlation is significant at the 0.01 level (2-tailed).

Results of the follow-up stepwise regression are summarized in Table 4. The analysis suggested that two predictors (i.e., depression and stress) contributed to psychological wellbeing. Depression explained 8% (*F*_1_, 918 = 81.66, *p* < 0.001), and stress explained an additional 2% (*F*_2_, 917 = 52.56, *p* < 0.001) of the variance in psychological wellbeing, with the regression formula *Y* = 50.08–0.58 + 0.33. Hence, Hypothesis 1 was supported.

**Table 4:** Stepwise multiple regression of predictors of psychological wellbeing (only significant predictors are shown).

Variable	*r* ^2^	*B*	SE	*β*	*T*
Depression	0.08	−0.58	0.07	−0.53	−8.69^**^
Stress	0.02	0.33	0.07	0.28	4.65^**^

** Correlation is significant at the 0.01 level (2-tailed).

### Testing Moderation Effect of Emotions

The PROCESS macro method (Model 1) was used to further analyze the moderation effect of emotions. Before conducting the analysis, all the variables were mean centered to minimize multicollinearity, and the results are summarized in Table 5.

**Table 5:** Testing the moderation effect of positive emotions on the relationship between depression, anxiety, and stress on psychological wellbeing.

	*b* (coefficient)	SE	*t*
Constant			
Depression	16.04	2.41	6.67^***^
Anxiety	16.11	2.54	6.35^***^
Stress	15.87	2.60	6.11^***^
Depression	0.04	0.09	0.50
Anxiety	0.02	0.10	0.16
Stress	0.01	0.10	0.12
Interaction			
Depression × Positive emotion	−0.01	0.00	−2.46^**^
Anxiety × Positive emotion	−0.01	0.00	−1.99^**^
Stress × Positive emotion	−0.01	0.00	−1.50

*** *p* < 0.000;

** *p* < 0.05.

**Figure 2:**
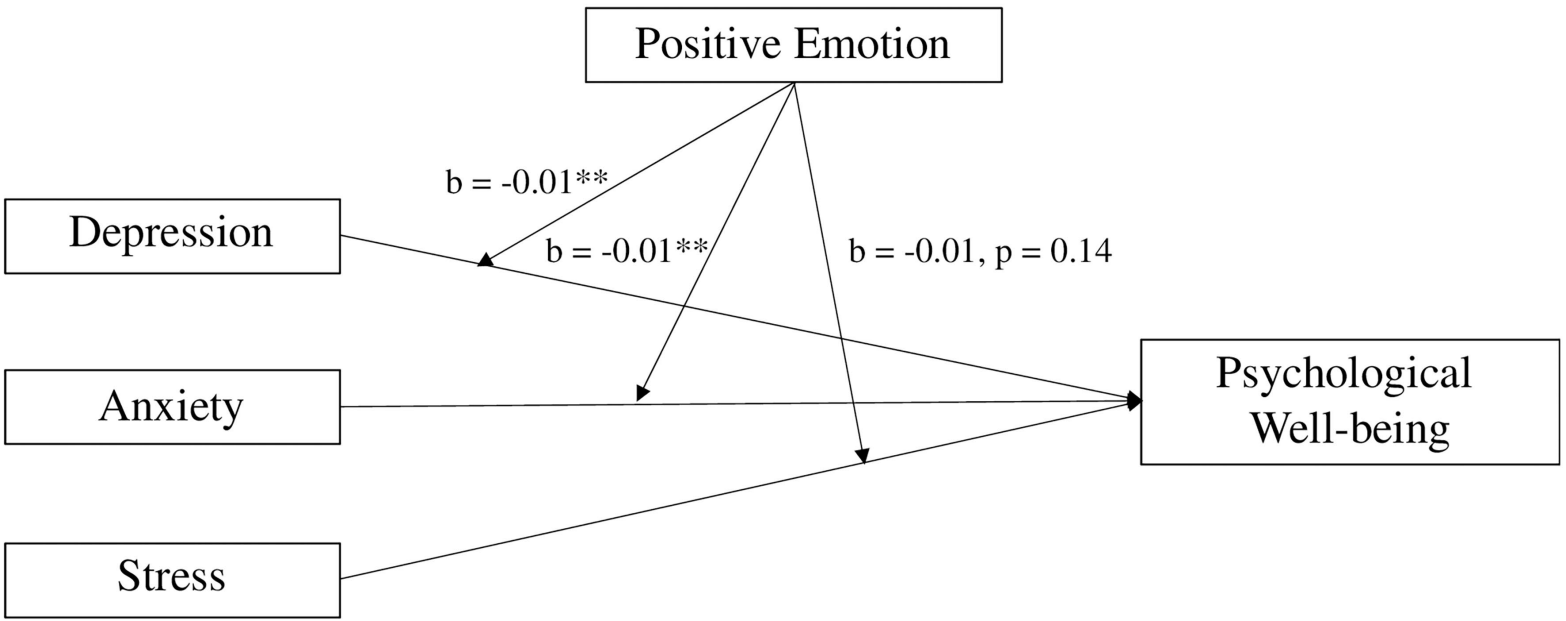
Positive emotion moderates the effect of the psychological outcomes (depression, anxiety, and stress) on psychological wellbeing. Note: ^**^p < 0.05.

The model indicates that positive emotion was positively related to psychological wellbeing (*b* = 1.07, *p* < 0.000), while depression was not (*b* = 0.04, *p* = 0.621). Nonetheless, the interaction term between positive emotion and depression was negatively related to psychological wellbeing (*b* = −0.01, *p* = 0.01). This trend was reflected in the interaction term between anxiety and positive emotion (*b* = −0.01, *p* = 0.05), but not between stress and positive emotion (*b* = −0.01, *p* = 0.14). The results are summarized in Figure 2.

Further analysis indicated that negative emotion did not have any significant moderating effect on the relationship between depression, anxiety, and stress with psychological wellbeing (see Table 6). Thus, Hypothesis 2 was partially supported.

**Table 6:** The moderation effect of negative emotion on the relationship between depression, anxiety, and stress on psychological wellbeing.

	*b* (coefficient)	SE	*t*
Constant			
Depression	56.49	2.90	19.51^***^
Anxiety	54.41	3.05	17.84^***^
Stress	50.55	3.11	16.26^***^
Depression	−0.38	0.13	−2.99^**^
Anxiety	−0.24	0.14	−1.67
Stress	0.00	0.13	0.00
Interaction			
Depression × Negative emotion	0.00	0.00	1.00
Anxiety × Negative emotion	0.00	0.01	0.36
Stress × Negative emotion	−0.00	0.00	−0.75

*** *p* < 0.00;

** *p* < 0.05.

### Testing Mediation Effect of Resilience

Similarly, the PROCESS macro (Model 4) in SPSS was used to perform the bootstrap method to test the direct and indirect effect (Preacher and Hayes, 2008) of resilience on psychological wellbeing (see Table 7).

**Table 7:** Indirect effect of depression, anxiety, and stress on psychological wellbeing, as mediated by resilience.

Dependent variable	Predictor	Mediator	*b*	95% C.I.	
				LLCI	ULCI
Psychological wellbeing	Depression Anxiety Stress	Resilience	−0.18	−0.22	−0.15	−0.19	−0.23	−0.15	−0.17	−0.21	−0.13

**Figure 3:**
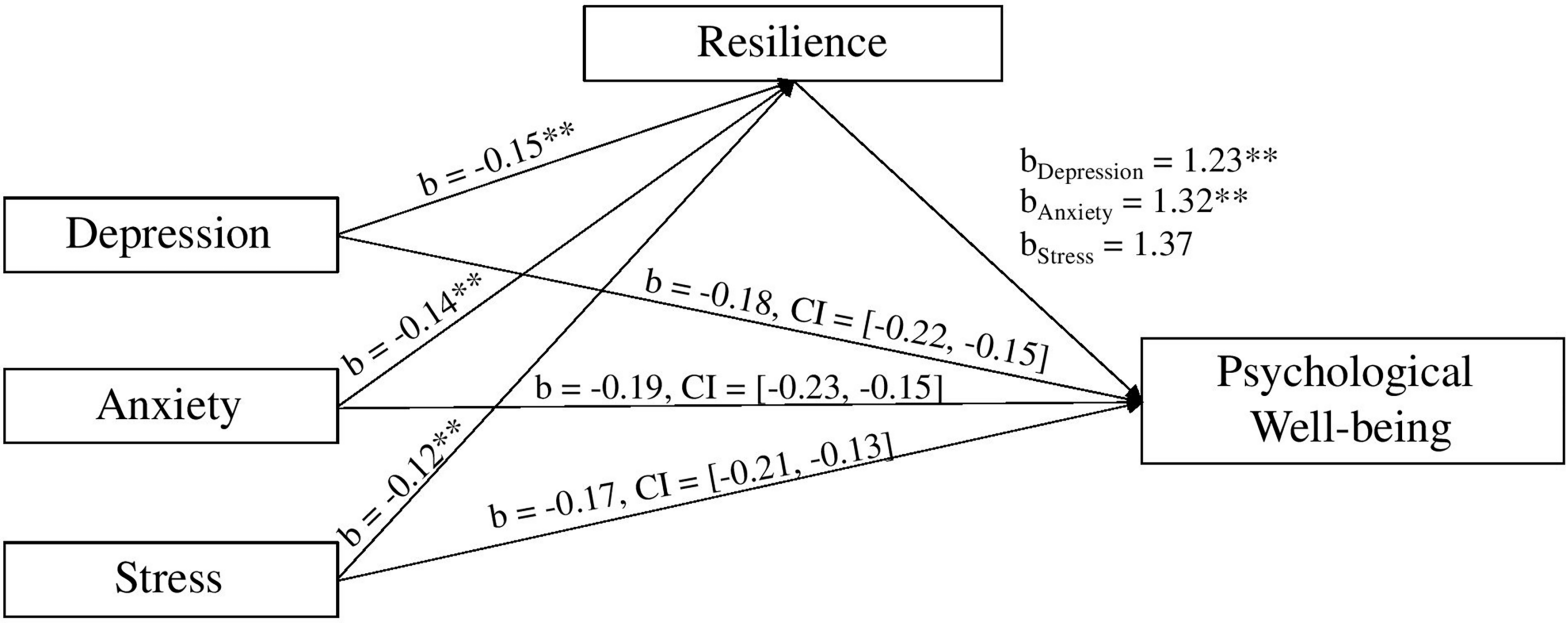
Resilience mediates the effect of the psychological outcomes (depression, anxiety, and stress) on psychological wellbeing. Note: ^**^p < 0.001.

The direct path coefficient from depression to psychological wellbeing was significant (*b* = −0.13, *t* = −3.69, *p* < 0.001), as was anxiety (*b* = −0.09, *t* = −2.31, *p* < 0.05). However, this was not the case for stress (*b* = −0.03, *t* = −0.80, *p* = 0.42). When resilience was included in the model, the indirect paths remained significant at a 95% confidence interval for depression [*b* = −0.18, 95% CI = (−0.22, −0.15)], anxiety [*b* = −0.19, 95% CI = (−0.23, −0.15)], and stress [*b* = −0.17, 95% CI = (−0.21, −0.12)]. These results are summarized in Figure 3.

These results reveal that, as expected, higher levels of depression, anxiety, and stress predicted significantly lower levels of resilience, and greater levels of resilience was associated with higher levels of psychological wellbeing. This effect persisted when controlling for resilience. This finding suggests that resilience did, like coping self-efficacy, explain the relationship between psychological outcomes and psychological wellbeing. Hence, Hypothesis 3 was supported.

### Testing Mediation Effect of Coping Self-Efficacy

The PROCESS macro (Model 4) in SPSS was used to perform the bootstrap method to test the direct and indirect effect (Preacher and Hayes, 2008) of coping self-efficacy on psychological wellbeing (see Table 8).

**Table 8:** Indirect effect of depression, anxiety, and stress on psychological wellbeing, as mediated by coping self-efficacy.

Dependent variable	Predictor	Mediator	*b*	95% CI	
				LLCI	ULCI
Psychological wellbeing	Depression	Coping self-efficacy	−0.08	−0.11	−0.00
	Anxiety		−0.07	−0.11	−0.03
	Stress		−0.04	−0.08	−0.00

**Figure 4:**
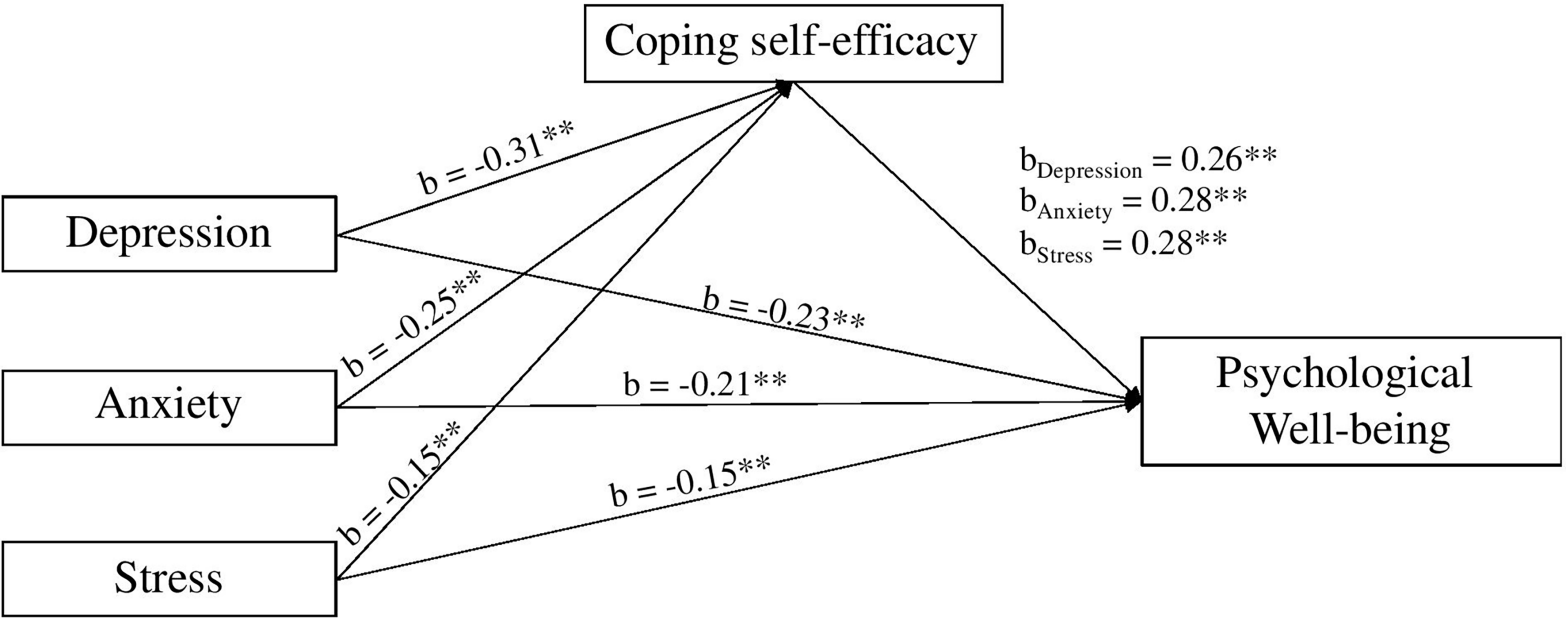
Coping self-efficacy mediates the effect of the psychological outcomes (depression, anxiety, and stress) on psychological wellbeing. Note: ^**^p < 0.001.

The direct path coefficient from depression to psychological wellbeing was significant (*b* = −0.23, *t* = −7.45, *p* < 0.000), as was anxiety (*b* = −0.21, *t* = −5.96, *p* < 0.000), and stress (*b* = −0.15, *t* = −4.66, *p* < 0.000). When coping self-efficacy was included in the model, the indirect paths remained significant for depression [*b* = −0.08, 95% CI = (−0.12, −0.05)], anxiety [*b* = −0.07, 95% CI = (−0.11, −0.03)], and stress [*b* = −0.04, 95% CI = (−0.08, −0.00); excluding 0]. These results are summarized in Figure 4.

These results indicated that, as expected, higher levels of depression, anxiety, and stress, significantly lowered levels of coping self-efficacy, and greater levels of coping self-efficacy was associated with higher levels of psychological wellbeing. The effect of depression, anxiety, and stress on psychological wellbeing remained significant after coping self-efficacy was added to the model. Hence, hypothesis 4 was supported. Table 9 summarizes the hypotheses tested and the results.

**Table 9:** Summary of hypotheses.

Hypotheses	
H1: Depression, anxiety, and stress associates negatively with psychological wellbeing	Supported
H2: Emotion moderates the relationship between depression, anxiety, and stress with psychological wellbeing	Partially supported with positive emotion as significant moderator
H3: Resilience mediates the relationship between depression, anxiety, and stress with psychological wellbeing	Supported
H4: Coping self-efficacy mediates the relationship between depression, anxiety, and stress with psychological wellbeing	Supported

## Discussion

The results indicated that the study’s established hypotheses had been fulfilled. The present findings regarding depression, anxiety, and stress were consistent with those conducted in other countries affected by COVID-19 (Cao et al., 2020; Zhang et al., 2020). A meta-analysis by Salari et al. (2020) provided substantial corroboration for a relationship between depression, anxiety, and stress with the psychological wellbeing of the general population during the COVID-19 pandemic. Sources of depression, anxiety, and stress can arise from the economic and social impact of COVID-19, which can, and has, led to the loss of income, isolation from family and friends, and fear of the future.

In line with past research, the moderation of depression, anxiety, and stress with psychological wellbeing by positive emotion is largely corroborated with Yıldırım and Güler’s (2021) study on the impact of positivity on distress related to COVID-19. The results are further substantiated by Seligman’s (2011) emphasis on the importance of positive emotion in the PERMA model as well as Yamaguchi et al.’s (2020) review on the association between positive emotion and the maintenance of mental health during COVID-19. It can be inferred then that contrary to previous literature, increasing positive emotion in this context would be an appropriate aspect to consider when developing interventions to maintain motivation and ensure the completion of tasks (Yih et al., 2020).

Moreover, it can be surmised from the study that the impact of depression, anxiety, and stress on the participant’s psychological wellbeing was ameliorated by their levels of resilience. This promising result reflects that by Wolmer et al. (2011), who discovered that individuals with high levels of resilience were capable of maintaining a state of functionality despite the challenges faced. Likewise, Riehm et al. (2021) further qualified this view with the longitudinal findings that participants with high levels of resilience reported no change in mental distress, while those who reported low to normal levels of resilience experienced a significant increase in mental distress. This pattern is indicative of the prominent role proactive or reactive resilience training may have during a high-stress public crisis (Kimhi, 2016; Song et al., 2020).

Lastly, the potential of coping self-efficacy as an effective protective factor against maladaptive coping behavior complementary to resilience has been established in this study, as supported by Zhou et al.’s (2021) study on self-efficacy and mental health problems. By improving one’s confidence, it bolsters one’s ability to overcome challenges, followed by a reduction in acute negative psychological response toward stressors. Learning these three elements not only benefits in the context of the present COVID-19 crisis, but also in long-term benefits by proactively preparing individuals for any further crisis that may occur in their life.

### Implications for Theory and Research

**Figure 5:**
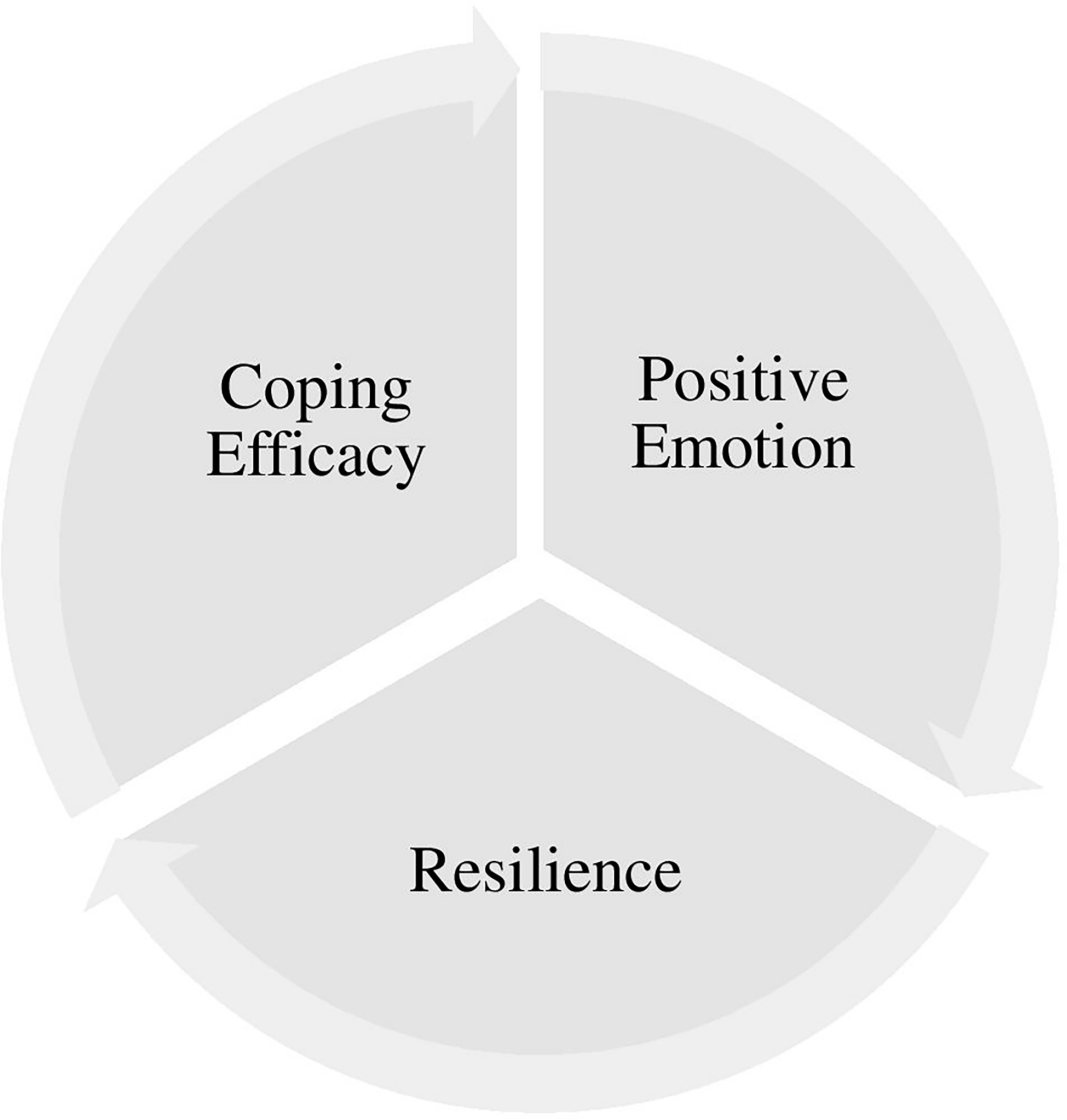
Positive emotion-resilience-coping efficacy (PERCE) model.

The COVID-19 pandemic has had a significant psychological impact on many professions especially frontlines such as healthcare workers (Gilleen et al., 2021). Existing theories of human flourishing and coping efficacy are too broad and general if used to address COVID-19 unprecedented mental health challenges. For example, Seligmen’s PERMA theory of wellbeing is an attempt to answer fundamental issues with five building blocks that enable flourishing – Positive Emotion, Engagement, Relationships, Meaning, and Accomplishment. In this study, the authors only used one of the five elements (positive emotion) and combined it with resilience and coping self-efficacy. Having established a general positive psychology model, it is possible to consider a dedicated model for the COVID-19 pandemic. Hence, this study results evidenced three key elements, namely positive emotion, resilience and coping self-efficacy, significantly mitigate the mental health challenges under the COVID-19 pandemic (Figure 5).

The Positive Emotion-Resilience-Coping Efficacy (PERCE) Model for COVID-19, is an integrated model developed from positive psychology and coping self-efficacy theories. These three key elements not only foster and promote psychological wellbeing but also act as an antecedent to living a happier life under the COVID-19 pandemic. With the growing mental health issues and urgent needs, researchers and practitioners are highly encouraged to develop intervention programs based on the PERCE Model for individuals who underwent adverse situations brought on by the Covid-19 pandemic, such as retrenchment, loss of job or income, working from home and others. Through the learning and development of positive emotion, resilience and coping self-efficacy, it improves not only the confidence of an individual to face adversity but increases the likelihood of an adaptive response toward negative stressors (Horn et al., 2016; Ye et al., 2020).

### Implications for Practice

The practical implications of these results are substantial when considering positive psychological interventions. At least three implications can be drawn from this study: First, it extended present COVID-19 research by providing foundational data regarding the psychological wellbeing of Malaysians. This potentially assists other South East Asian countries when they intend to compare the impact of COVID-19 on their population and perhaps strategize long-term recovery plans such as mental health interventions incorporating positive psychology and coping efficacy approaches using the PERCE Model; Second, the findings create public awareness regarding the current psychological wellbeing among Malaysians in the aftermath of COVID-19, emphasizing the importance of psychological support to local decision-makers. For example, ministries and government agencies should take a lead role in creating public awareness. Such initiatives include media, social media and online education advertisements and programs. Lastly, the outcomes presented scientific evidence to encourage the development of resilience, coping self-efficacy and positive emotion-focused interventions by professional mental health practitioners to assist the public. In particular, potentially, a ‘Global COVID-19 Psychology Consortium’ could be created with countries representative of professional mental health practitioners, across the globe. The expertise in this consortium could design intervention programs using the PERCE Model. Subsequently, these intervention programs could then be implemented in participative countries and help validate the model in diverse and large populations, with a hope to establish a ‘*PSYCHOLOGY VACCINE*’ Immunization Program.

For the practical significance of this study, the Positive Emotion-Resilience-Coping Efficacy (PERCE) model, proposed here, describes a recovery theory that potentially ameliorates the negative impact of COVID-19 on psychological wellbeing. This theory focuses on the enhancement of positive emotion, resilience, and coping self-efficacy to reduce maladaptive coping behavior and alleviate psychological distress. Drawing on existing theories, the proposed model shows how a combination of the described factors could produce adaptive coping behavior and improve wellbeing. Given that the presence of COVID-19 is unlikely to dissipate soon, it would be more pertinent to educate individuals on how to protect and enhance their own psychological wellbeing in the face of imminent challenges. Firstly, this study makes the case that the PERCE model has the potential to be further developed into ‘Psychological Intervention Programs’ that would have long-term benefits and aid individuals who are affected. In addition, for future study, an experimental design is proposed to examine the efficiency and effectiveness of the PERCE model. Secondly, the authors suggest that mental health practitioners such as clinical psychologists, coaching psychologists, counselors and others adopt the PERCE model into their practices to further enhance psychological wellbeing of their patients and clients. Lastly, COVID-19 is a global challenge, hence the authors also suggest that the PERCE model be tested across the globe with cultural and demographically diverse populations.

### Limitations and Recommendations for Future Research

When interpreting the findings, a few limitations should be considered. Firstly, the participants were volunteers from the public, with different demographic backgrounds and experience, therefore the findings may not apply to other specific groups of population in Malaysia.

Secondly, the research was conducted in Malaysia only and the findings may not apply to the affected population in another country. Thirdly, a survey with a cross-sectional design was used in this study which limits the in-depth information on participant’s experience.

Four recommendations can be derived from the above for future research. Firstly, with the presented evidence of potentially protective factors, an effective psychological intervention framework that integrates elements of positive and coaching psychology could be developed for testing (Song et al., 2020). Secondly, these results are unique to Malaysians, making it difficult to generalize them to South East Asians as a whole. However, this study does provide an avenue for replicability, to assess the veracity of what has been hypothesized, and how it can be compared to other countries under similar conditions. Thirdly, future research could apply an alternative study design such as interview and psychological intervention to validate the supported hypotheses. Lastly, future research could design intervention programs using the PERCE Model, and subsequently validate this model under the COVID-19 pandemic or another similar situation.

## Conclusion

From this, it can be concluded that the findings evidenced positive emotion, resilience and coping self-efficacy are fundamental elements in managing psychological wellbeing affected by COVID-19. Future research recommendations include designing psychological interventions using the PERCE model and further validating those intervention programs. In summary, this study not only builds a new positive psychology model for the COVID-19 pandemic but also contributes to the psychological recovery phase.

## Data Availability Statement

The raw data supporting the conclusions of this article will be made available by the authors, without undue reservation.

## Ethics Statement

The studies involving human participants were reviewed and approved by Ethics Committee at School of Social Sciences, Heriot-Watt University. The patients/participants provided their written informed consent to participate in this study.

## Author Contributions

GK and DG conceptualized the study and were actively and equally involved in all stages of the research, including funding application, ethics application, designing of study, data collection, data analysis, and presentation of results. GK, DG, and RW equally contributed toward manuscript writing, editing, and proof-reading. RW also contributed to the data collection and data analysis stage of the research. SC contributed to editing and proof-reading. RK contributed to the selection of survey questionnaires and variables, as well as proof-reading in the initial stage of the research. WL contributed to the data analysis stage of the research. KK contributed to the data collection stage of the research. All authors contributed to the article and approved the submitted version.

## Funding

This research was funded by Global Challenges Research Fund, The Scottish Funding Council, grant number SFC: P20GCRF7.

## Conflict of Interest

The authors declare that the research was conducted in the absence of any commercial or financial relationships that could be construed as a potential conflict of interest.

## Publisher’s Note

All claims expressed in this article are solely those of the authors and do not necessarily represent those of their affiliated organizations, or those of the publisher, the editors and the reviewers. Any product that may be evaluated in this article, or claim that may be made by its manufacturer, is not guaranteed or endorsed by the publisher.

## References

[ref1] Ascenso S. Perkins R. Williamon A. (2018). Resounding meaning: a PERMA wellbeing profile of classical musicians. Front. Psychol. 9, 1895. doi: 10.3389/fpsyg.2018.01895, PMID: 30459665 PMC6232231

[ref2] Asmundson G. J. Taylor S. (2020). How health anxiety influences responses to viral outbreaks like COVID-19: what all decision-makers, health authorities, and health care professionals need to know. J. Anxiety Disord. 71:102211. doi: 10.1016/j.janxdis.2020.102211, PMID: 32179380 PMC7271220

[ref3] Asselmann E. Specht J. (2020). Taking the ups and downs at the rollercoaster of love: associations between major life events in the domain of romantic relationships and the big five personality traits. Dev. Psychol. 56, 1803–1816. doi: 10.1037/dev0001047, PMID: 32672996

[ref4] Bandura A. (1982). Self-efficacy mechanism in human agency. Am. Psychol. 37, 122–147. doi: 10.1037/0003-066X.37.2.122, PMID: 32811480

[ref5] Barzilay R. Moore T. M. Greenberg D. M. DiDomenico G. E. Brown L. A. White L. K.. (2020). Resilience, COVID-19-related stress, anxiety and depression during the pandemic in a large population enriched for healthcare providers. Transl. Psychiatry 10, 1–8. doi: 10.1038/s41398-020-00982-4 32820171 PMC7439246

[ref6] Benke C. Autenrieth L. K. Asselmann E. Pané-Farré C. A. (2020). Stay-at-home orders due to the COVID-19 pandemic are associated with elevated depression and anxiety in younger, but not older adults: results from a nationwide community sample of adults from Germany. Psychol. Med. 1–2. doi: 10.1017/S0033291720003438, PMID: 32895064 PMC7487742

[ref7] Bidzan M. Bidzan-Bluma I. Szulman-Wardal A. Stueck M. Bidzan M. (2020). Does self-efficacy and emotional control protect hospital staff from COVID-19 anxiety and PTSD symptoms? Psychological functioning of hospital staff after the announcement of COVID-19 coronavirus pandemic. Front. Psychol. 11. doi: 10.3389/fpsyg.2020.552583, PMID: 33424673 PMC7785971

[ref8] Bolton K. W. Hall J. C. Blundo R. Lehmann P. (2017). The role of resilience and resilience theory in solution-focused practice. J. Syst.Therap. 36, 1–15. doi: 10.1521/jsyt.2017.36.3.1

[ref9] Brooks S. K. Webster R. K. Smith L. E. Woodland L. Wessely S. Greenberg N. (2020). i Rubin, GJ (2020). The psychological impact of quarantine and how to reduce it: rapid review of the evidence. Lancet, 395, 912–920, doi: 10.1016/S0140-6736(20)30460-8, PMID: 32112714 PMC7158942

[ref11] Cao W. Fang Z. Hou G. Han M. Xu X. Dong J.. (2020). The psychological impact of the COVID-19 epidemic on college students in China. Psychiatry Res. 287, 112934. doi: 10.1016/j.psychres.2020.112934, PMID: 32229390 PMC7102633

[ref12] Chen Y. Peng Y. Xu H. O‟Brien W. H. (2018). Age differences in stress and coping: problem-focused strategies mediate the relationship between age and positive affect. Int. J. Aging Hum. Dev. 86, 347–363. doi: 10.1177/0091415017720890, PMID: 28789561

[ref13] Chesney M. A. Neilands T. B. Chambers D. B. Taylor J. M. Folkman S. (2006). A validity and reliability study of the coping self-efficacy scale. Br. J. Health Psychol. 11, 421–437. doi: 10.1348/135910705X53155, PMID: 16870053 PMC1602207

[ref14] Cheung C. K. Tse J. W. L. (2008). Institutional trust as a determinant of anxiety during the SARS crisis in Hong Kong. Soc. Work Public Health 23, 41–54. doi: 10.1080/19371910802053224, PMID: 19301537

[ref15] Czeisler M. É. Lane R. I. Petrosky E. Wiley J. F. Christensen A. Njai R.. (2020). Mental health, substance use, and suicidal ideation during the COVID-19 pandemic—United States, June 24–30, 2020. Morb. Mortal. Wkly Rep. 69, 1049–1057. doi: 10.15585/mmwr.mm6932a1, PMID: 32790653 PMC7440121

[ref16] Czymara C. S. Langenkamp A. Cano T. (2021). Cause for concerns: gender inequality in experiencing the COVID-19 lockdown in Germany. Eur. Soc. 23, S68–S81. doi: 10.1080/14616696.2020.1808692

[ref17] Dang H. A. H. Nguyen C. V. (2021). Gender inequality during the COVID-19 pandemic: income, expenditure, savings, and job loss. World Dev. 140:105296. doi: 10.1016/j.worlddev.2020.105296, PMID: 34548740 PMC8446715

[ref18] Daniel W. W. (1999). Biostatistics: A Foundation for Analysis in the Health Sciences. 7th *Edn*. New York, John Wiley and Sons Inc.

[ref19] Elcheroth G. Drury J. (2020). Collective resilience in times of crisis: lessons from the literature for socially effective responses to the pandemic. Br. J. Soc. Psychol. 59, 703–713. doi: 10.1111/bjso.12403, PMID: 32584480 PMC7361591

[ref20] Emdin C. A. Odutayo A. Wong C. X. Tran J. Hsiao A. J. Hunn B. H. (2016). Meta-analysis of anxiety as a risk factor for cardiovascular disease. Am. J. Cardiol. 118, 511–519. doi: 10.1016/j.amjcard.2016.05.041, PMID: 27324160

[ref21] Farmer N. Cotter E. W. (2021). Well-being and cooking behavior: using the positive emotion, engagement, relationships, meaning, and accomplishment (PERMA) model as a theoretical framework. Front. Psychol. 12, 1196. doi: 10.3389/fpsyg.2021.560578 PMC8071848 33912092

[ref22] Farré L. Fawaz Y. González L. Graves J. (2020). How the COVID-19 Lockdown Affected Gender Inequality in Paid and Unpaid Work in Spain. [Preprint]. doi: 10.2139/ssrn.3643198

[ref23] Faudzi F. N. M. Armitage C. J. Bryant C. Brown L. J. (2019). Moderating effects of age on relationships between attitudes to aging and well-being outcomes. Aging Ment. Health 16, 1–7. doi: 10.1080/13607863.2019.1619167 31099260

[ref24] Folkman S. Lazarus R. S. (1984). Stress, Appraisal, and Coping. New York: Springer Publishing Company.

[ref25] Forgeard M. J. Jayawickreme E. Kern M. L. Seligman M. E. (2011). Doing the right thing: measuring wellbeing for public policy. Int. J. Wellbeing 1. doi: 10.5502/ijw.v1i1.15

[ref001] Gilleen J. Santaolalla A. Valdearenas L. Salice C. Fusté M. (2021). Impact of the COVID-19 pandemic on the mental health and well-being of UK healthcare workers. BJPsych Open 7. 10.1192/bjo.2021.42 PMC8082128 33910674

[ref26] Grajfoner D. Ke G. N. Wong R. M. M. (2021). The effect of pets on human mental health and wellbeing during COVID-19 lockdown in Malaysia. Animals 11, 2689. doi: 10.3390/ani11092689, PMID: 34573655 PMC8470955

[ref27] Grant L. Kinman G. (2014). Emotional resilience in the helping professions and how it can be enhanced. Health Soc. Care Educ. 3, 23–34. doi: 10.11120/hsce.2014.00040

[ref28] Hawryluck L. Gold W. L. Robinson S. Pogorski S. Galea S. Styra R. (2004). SARS control and psychological effects of quarantine, Toronto. Can. Emerg. infect. Dis. 10, 1206–1212. doi: 10.3201/eid1007.030703, PMID: 15324539 PMC3323345

[ref29] Hayes A. F. Montoya A. K. Rockwood N. J. (2017). The analysis of mechanisms and their contingencies: PROCESS versus structural equation modeling. Australas. Mark. J. 25, 76–81. doi: 10.1016/j.ausmj.2017.02.001

[ref30] Henry J. D. Crawford J. R. (2005). The short-form version of the depression anxiety stress scales (DASS-21): construct validity and normative data in a large non-clinical sample. Br. J. Clin. Psychol. 44, 227–239. doi: 10.1348/014466505X29657, PMID: 16004657

[ref31] Horn S. R. Charney D. S. Feder A. (2016). Understanding resilience: new approaches for preventing and treating PTSD. Exp. Neurol. 284, 119–132. doi: 10.1016/j.expneurol.2016.07.002, PMID: 27417856

[ref32] Jamaludin S. Azmir N. A. Ayob A. F. M. Zainal N. (2020). COVID-19 exit strategy: transitioning towards a new normal–review article. Ann. Med. Surg. 59, 165–170. doi: 10.1016/j.amsu.2020.09.046 PMC7529589 33024558

[ref33] Kimhi S. (2016). Levels of resilience: associations among individual, community, and national resilience. J. Health Psychol. 21, 164–170. doi: 10.1177/1359105314524009, PMID: 24594483

[ref34] Lades L. K. Laffan K. Daly M. Delaney L. (2020). Daily emotional well-being during the COVID-19 pandemic. Br. J. Health Psychol. 25, 902–911. doi: 10.1111/bjhp.12450, PMID: 32573074 PMC7361840

[ref35] Lazzarino A. I. Yiengprugsawan V. Seubsman S. A. Steptoe A. Sleigh A. C. (2014). The associations between unhealthy behaviours, mental stress, and low socio-economic status in an international comparison of representative samples from Thailand and England. Glob. Health 10, 10–18. doi: 10.1186/1744-8603-10-10, PMID: 24555674 PMC3933467

[ref36] Lee J. H. Nam S. K. Kim A. Kim B. Lee M. Y. Lee S. M. (2013). Resilience: a meta-analytic approach. J. Couns. Dev. 91, 269–279. doi: 10.1002/j.1556-6676.2013.00095.x, PMID: 35127038

[ref37] Li S. Wang Y. Xue J. Zhao N. Zhu T. (2020). The impact of COVID-19 epidemic declaration on psychological consequences: a study on active Weibo users. Int. J. Environ. Res. Public Health 17:2032. doi: 10.3390/ijerph17062032, PMID: 32204411 PMC7143846

[ref38] Lip H. T. C. Huei T. J. Mohamad Y. Alwi R. I. Mat T. N. A. T. (2020). Critical adjustments and trauma surgery trends in adaptation to COVID-19 pandemic in Malaysia. Chinese J. Traumatol. 23, 207–210. doi: 10.1016/j.cjtee.2020.05.007, PMID: 32653358 PMC7255157

[ref39] Liu H. Zhang C. Ji Y. Yang L. (2018). Biological and psychological perspectives of resilience: is it possible to improve stress resistance? Front. Hum. Neurosci. 12, 326. doi: 10.3389/fnhum.2018.00326, PMID: 30186127 PMC6110926

[ref40] Longstaff P. H. Yang S. U. (2008). Communication management and trust: their role in building resilience to “surprises” such as natural disasters, pandemic flu, and terrorism. Ecol. Soc. 13. doi: 10.5751/ES-02232-130103

[ref41] Luchetti M. Lee J. H. Aschwanden D. Sesker A. Strickhouser J. E. Terracciano A.. (2020). The trajectory of loneliness in response to COVID-19. Am. Psychol. Adv. 75, 897–908. doi: 10.1037/amp0000690, PMID: 32567879 PMC7890217

[ref42] Minhat H. S. Kadir Shahar H. (2020). The trajectory of COVID-19 scenario in Malaysia: facing the unprecedented. Curr. Med. Res. Opin. 36, 1309–1311. doi: 10.1080/03007995.2020.1786680, PMID: 32569488

[ref43] Morera O. F. Stokes S. M. (2016). Coefficient α as a measure of test score reliability: review of 3 popular misconceptions. Am. J. Public Health 106, 458–461. doi: 10.2105/AJPH.2015.302993, PMID: 26885962 PMC4816140

[ref44] Naderipour A. Abdul-Malek Z. Ahmad N. A. Kamyab H. Ashokkumar V. Ngamcharussrivichai C.. (2020). Effect of COVID-19 virus on reducing GHG emission and increasing energy generated by renewable energy sources: a brief study in Malaysian context. Environ. Technol. Innov. 20, 101151. doi: 10.1016/j.eti.2020.101151, PMID: 32923529 PMC7480331

[ref45] Ng S. M. Ke G. N. Wilks R. (2014). The mediating role of work locus of control on the relationship among emotional intelligence, organisational citizenship behaviours, and mental health among nurses. Aust. J. Psychol. 66, 207–215. doi: 10.1111/ajpy.12049

[ref46] Nooraie R. Y. Warren K. Juckett L. A. Cao Q. A. Bunger A. C. Patak-Pietrafesa M. A. (2021). Individual-and group-level network-building interventions to address social isolation and loneliness: a scoping review with implications for COVID19. PLoS One 16:e0253734. doi: 10.1371/journal.pone.0253734, PMID: 34170980 PMC8232435

[ref47] Oei T. P. Sawang S. Goh Y. W. Mukhtar F. (2013). Using the depression anxiety stress scale 21 (DASS-21) across cultures. Int. J. Psychol. 48, 1018–1029. doi: 10.1080/00207594.2012.755535, PMID: 23425257

[ref48] Omar S. Z. Zaremohzzabieh Z. Samah A. A. Bolong J. Shaffril H. A. M. (2020). Influence of different facets of internet addiction on subjective well-being in Malaysia: a comparison across ethnic groups. J. Komun. Malays. J. Commun. 36, 196–211. doi: 10.17576/JKMJC-2020-3602-12

[ref49] Palmer S. Panchal S. O’Riordan S. (2020). Could the experience of the COVID-19 pandemic have any positive impact on wellbeing? Eur. J. Appl. Posit. Psychol. 4, 2397–7116.

[ref50] Pieh C. Budimir S. Probst T. (2020). The effect of age, gender, income, work, and physical activity on mental health during coronavirus disease (COVID-19) lockdown in Austria. J. Psychosom. Res. 136:110186. doi: 10.1016/j.jpsychores.2020.110186, PMID: 32682159 PMC7832650

[ref51] Preacher K. J. Hayes A. F. (2008). Asymptotic and resampling strategies for assessing and comparing indirect effects in multiple mediator models. Behav. Res. Methods 40, 879–891. doi: 10.3758/BRM.40.3.879, PMID: 18697684

[ref52] Prime H. Wade M. Browne D. T. (2020). Risk and resilience in family well-being during the COVID-19 pandemic. Am. Demogr. Psychologist 75, 631–643. doi: 10.1037/amp0000660 32437181

[ref53] Restubog S. L. D. Ocampo A. C. G. Wang L. (2020). Taking control amidst the chaos: emotion regulation during the COVID-19 pandemic. J. Vocat. Behav. 119:103440. doi: 10.1016/j.jvb.2020.103440, PMID: 32390659 PMC7206430

[ref54] Riehm K. E. Brenneke S. G. Adams L. B. Gilan D. Lieb K. Kunzler A. M.. (2021). Association between psychological resilience and changes in mental distress during the COVID-19 pandemic. J. Affect. Disord. 282, 381–385. doi: 10.1016/j.jad.2020.12.071, PMID: 33421866 PMC7889692

[ref55] Robertson I. T. Cooper C. L. Sarkar M. Curran T. (2015). Resilience training in the workplace from 2003 to 2014: a systematic review. J. Occup. Organ. Psychol. 88, 533–562. doi: 10.1111/joop.12120

[ref56] Sahni P. S. Singh K. Sharma N. Garg R. (2021). Yoga an effective strategy for self-management of stress-related problems and wellbeing during COVID19 lockdown: a cross-sectional study. PLoS One 16:e0245214. doi: 10.1371/journal.pone.0245214, PMID: 33566848 PMC7875402

[ref57] Salari N. Hosseinian-Far A. Jalali R. Vaisi-Raygani A. Rasoulpoor S. Mohammadi M.. (2020). Prevalence of stress, anxiety, depression among the general population during the COVID-19 pandemic: a systematic review and meta-analysis. Glob. Health 16, 1–11. doi: 10.1186/s12992-020-00589-w PMC7338126 32631403

[ref58] Seligman M. E. P. (2011). Flourish. New York: Free Press.

[ref59] Seligman M. (2018). PERMA and the building blocks of well-being. J. Posit. Psychol. 13, 333–335. doi: 10.1080/17439760.2018.1437466

[ref60] Seligman M. E. Csikszentmihalyi M. (2014). “Positive psychology: an introduction” in Flow and the Foundations of Positive Psychology (Dordrecht: Springer), 279–298. doi: 10.1007/978-94-017-9088-8_18

[ref61] Shah A. U. M. Safri S. N. A. Thevadas R. Noordin N. K. Abd Rahman A. Sekawi Z.. (2020). COVID-19 outbreak in Malaysia: actions taken by the Malaysian government. Int. J. Infect. Dis. 97, 108–116. doi: 10.1016/j.ijid.2020.05.093, PMID: 32497808 PMC7264933

[ref62] Smith B. W. Dalen J. Wiggins K. Tooley E. Christopher P. Bernard J. (2008). The brief resilience scale: assessing the ability to bounce back. Int. J. Behav. Med. 15, 194–200. doi: 10.1080/10705500802222972, PMID: 18696313

[ref63] Song Y. Swendiman R. A. Shannon A. B. Torres-Landa S. Khan F. N. Williams N. N.. (2020). Can we coach resilience? An evaluation of professional resilience coaching as a well-being initiative for surgical interns. J. Surg. Educ. 77, 1481–1489. doi: 10.1016/j.jsurg.2020.04.014, PMID: 32446771

[ref64] Streiner D. L. (2003). Starting at the beginning: an introduction to coefficient alpha and internal consistency. J. Pers. Assess. 80, 99–103. doi: 10.1207/S15327752JPA8001_18, PMID: 12584072

[ref65] Tang K. H. D. (2020). Movement control as an effective measure against Covid-19 spread in Malaysia: an overview. J. Public Health 30, 583–586. doi: 10.1007/s10389-020-01316-w PMC7293423 32837842

[ref66] Tennant R. Hiller L. Fishwick R. Platt S. Joseph S. Weich S.. (2007). The Warwick-Edinburgh mental well-being scale (WEMWBS): development and UK validation. Health Qual. Life Outcomes 5, 63. doi: 10.1186/1477-7525-5-63, PMID: 18042300 PMC2222612

[ref67] Tugade M. M. Fredrickson B. L. Feldman Barrett L. (2004). Psychological resilience and positive emotional granularity: examining the benefits of positive emotions on coping and health. J. Pers. 72, 1161–1190. doi: 10.1111/j.1467-6494.2004.00294.x, PMID: 15509280 PMC1201429

[ref68] Vinkers C. H. van Amelsvoort T. Bisson J. I. Branchi I. Cryan J. F. Domschke K.. (2020). Stress resilience during the coronavirus pandemic. Eur. Neuropsychopharmacol. 35, 12–16. doi: 10.1016/j.euroneuro.2020.05.003, PMID: 32446705 PMC7211573

[ref69] Waiho K. Fazhan H. Ishak S. D. Kasan N. A. Liew H. J. Norainy M. H.. (2020). Potential impacts of COVID-19 on the aquaculture sector of Malaysia and its coping strategies. Aquacult. Rep. 18:100450. doi: 10.1016/j.aqrep.2020.100450

[ref70] Wang Y. Jing X. Han W. Jing Y. Xu L. (2020). Positive and negative affect of university and college students during COVID-19 outbreak: a network-based survey. Int. J. Public Health 65, 1437–1443. doi: 10.1007/s00038-020-01483-3, PMID: 33063142 PMC7561237

[ref71] Watson D. Clark L. A. Tellegen A. (1988). Development and validation of brief measures of positive and negative affect: the PANAS scales. J. Pers. Soc. Psychol. 54, 1063–1070. doi: 10.1037/0022-3514.54.6.1063, PMID: 3397865

[ref72] Wheaton M. G. Abramowitz J. S. Berman N. C. Fabricant L. E. Olatunji B. O. (2012). Psychological predictors of anxiety in response to the H1N1 (swine flu) pandemic. Cogn. Ther. Res. 36, 210–218. doi: 10.1007/s10608-011-9353-3

[ref73] Wolmer L. Hamiel D. Barchas J. D. Slone M. Laor N. (2011). Teacher-delivered resilience-focused intervention in schools with traumatized children following the second Lebanon war. J. Trauma. Stress. 24, 309–316. doi: 10.1002/jts.20638, PMID: 21618288

[ref74] Yamaguchi K. Takebayashi Y. Miyamae M. Komazawa A. Yokoyama C. Ito M. (2020). Role of focusing on the positive side during COVID-19 outbreak: mental health perspective from positive psychology. Psychol. Trauma Theory Res. Pract. Policy 12, S49–S50. doi: 10.1037/tra0000807, PMID: 32525365

[ref75] Yang H. Ma J. (2020). How an epidemic outbreak impacts happiness: factors that worsen (vs. protect) emotional well-being during the coronavirus pandemic. Psychiatry Res. 289:113045. doi: 10.1016/j.psychres.2020.113045, PMID: 32388418 PMC7190485

[ref76] Ye Z. Yang X. Zeng C. Wang Y. Shen Z. Li X.. (2020). Resilience, social support, and coping as mediators between COVID-19-related stressful experiences and acute stress disorder among college students in China. Appl. Psychol. Health Well Being 12, 1074–1094. doi: 10.1111/aphw.12211, PMID: 32666713 PMC7405224

[ref77] Yih J. Kirby L. D. Smith C. A. (2020). Profiles of appraisal, motivation, and coping for positive emotions. Cognit. Emot. 34, 481–497. doi: 10.1080/02699931.2019.1646212, PMID: 32314674

[ref78] Yıldırım M. Güler A. (2021). Positivity explains how COVID-19 perceived risk increases death distress and reduces happiness. Personal. Individ. Differ. 168:110347. doi: 10.1016/j.paid.2020.110347, PMID: 32843780 PMC7439822

[ref79] Zhang S. X. Wang Y. Rauch A. Wei F. (2020). Unprecedented disruption of lives and work: health, distress and life satisfaction of working adults in China one month into the COVID-19 outbreak. Psychiatry Res. 288, 112958. doi: 10.1016/j.psychres.2020.112958, PMID: 32283450 PMC7146665

[ref80] Zhou C. Yue X. D. Zhang X. Shangguan F. Zhang X. Y. (2021). Self-efficacy and mental health problems during COVID-19 pandemic: a multiple mediation model based on the health belief model. Personal. Individ. Differ. 179:110893. doi: 10.1016/j.paid.2021.110893 PMC9756413 36540084

